# Extracellular Vesicle-Derived Spliced Leader RNA as a Minimally Invasive Molecular Biomarker for Leishmaniasis Detection

**DOI:** 10.3390/tropicalmed11100281

**Published:** 2026-10-03

**Authors:** Mohannasadat Sajjadi Oskouei, Juan P. Gonzalez, Delis J. Mattei-Lopez, Douglas A. Shoue, Cristian Koepfli, Mary Ann McDowell

**Affiliations:** 1 Department of Biological Sciences, University of Notre Dame, Notre Dame, IN 46556, USA; msajjadi@nd.edu (M.S.O.); jgonza34@nd.edu (J.P.G.); dmatteil@nd.edu (D.J.M.-L.); dshoue@nd.edu (D.A.S.); ckoepfli@nd.edu (C.K.); 2 Eck Institute for Global Health, University of Notre Dame, Notre Dame, IN 46556, USA

**Keywords:** visceral leishmaniasis, extracellular vesicles, spliced leader RNA, biomarker diagnostics, EV-based diagnosis

## Abstract

Leishmaniasis continues to be a major neglected tropical disease, particularly in endemic areas where reliable diagnosis remains challenging. Many existing diagnostic methods are invasive, lack sufficient sensitivity, or require equipment and expertise that may not be available in resource-limited settings. Parasite-derived spliced leader (SL) RNA has recently gained attention as a promising molecular marker because it may enable the sensitive detection of viable *Leishmania* parasites and help track changes in infection over time. Extracellular vesicles (EVs) are also of growing interest because they protect and transport stable biomolecules that may provide valuable diagnostic information and influence interactions between the parasite and its host. In this proof-of-concept study, we evaluated the feasibility of detecting *Leishmania*-derived SL-RNA in EV-enriched preparations from experimental leishmaniasis model systems. EV-enriched preparations were isolated from parasite-conditioned media (PCM), infected THP-1 macrophage (I-MP) cell-conditioned medium (CCM), and serum from experimentally infected mice using a precipitation method. As a complementary isolation approach, additional preparations were isolated from PCM by ultracentrifugation. An aliquot of each preparation was analyzed by nanoparticle tracking analysis to determine particle size and concentration, and the remaining portion was used for RNA extraction. RNA was reverse transcribed into cDNA and analyzed for SL-RNA amplification using quantitative polymerase chain reaction (qPCR). SL-RNA amplification was successfully detected in PCM, I-MP CCM, and serum from experimentally infected mice, while uninfected controls showed no specific amplification. These findings were independently confirmed via ddPCR analysis. Notably, EV-enriched preparations from infected mice yielded a stronger SL-RNA signal than serum alone, highlighting the diagnostic potential of targeting SL-RNA in EV-enriched human clinical samples, particularly in low-burden infections. Although these findings support the feasibility of EV-associated SL-RNA as a molecular biomarker for *Leishmania* infection, clinical validation with human samples is required.

## 1. Introduction

Leishmaniasis is a neglected tropical disease caused by protozoan parasites of the genus *Leishmania* and transmitted through the bite of infected female phlebotomine sand flies [1,2,3]. The disease remains a major global health burden, especially in tropical and subtropical regions where access to healthcare is often limited. It disproportionately affects vulnerable and underserved populations living in low-resource settings [4,5,6,7]. It is estimated that hundreds of thousands of new visceral leishmaniasis (VL) and cutaneous leishmaniasis (CL) cases occur annually, with disease endemicity reported across regions of Asia, Africa, South America, and the Mediterranean basin [8,9,10]. 

Despite advances in disease surveillance and treatment, accurate diagnosis of leishmaniasis remains a major clinical challenge [11]. Conventional parasitological methods, including microscopy, tissue biopsy, and splenic or bone marrow aspiration, remain important diagnostic approaches but are invasive, require skilled personnel, and often show reduced sensitivity in low-parasite-burden or chronic infections [12,13,14]. Serological assays such as recombinant K39 antigen-based rapid diagnostic test (rK39 RDT), indirect fluorescent antibody test (IFAT), enzyme-linked immunosorbent assay (ELISA), and direct agglutination test (DAT) provide less invasive alternatives for VL diagnosis. However, their sensitivity and specificity can vary depending on geographic region, infecting species, and host immune status [15,16,17]. Molecular methods such as polymerase chain reaction (PCR) and quantitative PCR (qPCR) have significantly improved analytical sensitivity and specificity for *Leishmania* detection [11]. However, DNA persistence following parasite death may limit their utility for monitoring active infection or treatment response [18].

Recently, RNA-based molecular targets have gained attention as indicators of viable *Leishmania* parasites. Among these targets, spliced leader RNA (SL-RNA) is of particular interest because of its conserved nature, high abundance, and association with metabolically active organisms [19]. SL-RNA, also known as mini-exon RNA, is a short non-coding RNA involved in the maturation of parasite mRNAs [20,21]. In *Leishmania*, genes are transcribed as long polycistronic transcripts, and mature mRNAs are generated through trans-splicing, during which the capped spliced leader sequence is added to the 5′ end of each mRNA [20,21]. The mini-exon/spliced leader region is highly conserved across *Leishmania* species but remains distinct enough from related kinetoplastid parasites to be useful as a pan-*Leishmania* detection target [22,23]. Because SL-RNA is abundant and linked to active parasite RNA expression, SL-RNA-based detection may offer a more sensitive and biologically relevant approach than DNA-based methods, especially for identifying active infection and monitoring treatment response [19]. However, direct RNA detection in blood or tissue requires appropriate sample preservation and extraction because RNA is susceptible to degradation [24,25]. 

Extracellular vesicles (EVs) are lipid bilayer membrane-delimited particles released by all cell types and contribute to intercellular communication through the transfer of proteins, lipids, metabolites, and nucleic acids [26,27]. EVs comprise heterogeneous populations that differ in their biogenesis, size, and molecular composition [26,28].

EVs play an important role in host-pathogen communication during infectious diseases by transporting virulence factors, immunomodulatory molecules, and regulatory RNAs that can alter immune responses and support pathogen survival [29,30,31,32]. In leishmaniasis, parasite-derived EVs and EV-enriched preparations have been shown to deliver parasite proteins to macrophages and to modulate macrophage signaling, cytokine production, and immune responses [33,34,35,36,37,38].

Enrichment of extracellular particles may help address the instability and low abundance of parasite RNA by concentrating particle-associated material and protecting RNA from degradation. Culture-based studies have shown that EV preparations from *L. donovani* and *L. braziliensis* contain selectively enriched parasite-derived small RNAs and that intact vesicles protect this RNA cargo from RNase-mediated degradation [39]. Evidence from schistosomiasis also indicates that enrichment of serum extracellular particles can improve the recovery and detection of low-abundance parasite-derived RNAs compared with direct RNA extraction from serum [40]. Similarly, tRNA- and rRNA-derived fragments have been detected in EV-enriched plasma preparations from mice infected with *L. donovani* or *L. amazonensis*, suggesting that parasite RNA can circulate during infection [41]. In patients with VL, *Leishmania*-derived proteins have been identified in plasma EV preparations; however, evidence for parasite-derived RNA in human samples remains limited [33]. 

The studies described above did not investigate whether SL-RNA is present in EV-enriched preparations from infected host cells or circulating biofluids. It also remains unclear whether SL-RNA recovered after particle enrichment is enclosed within membrane-bound vesicles or co-isolated with non-vesicular material [26]. 

We hypothesized that *Leishmania*-derived SL-RNA could be detected in EV-enriched preparations obtained from PCM, I-MP CCM, and serum from experimentally infected mice. Therefore, this proof-of-concept study aimed to evaluate the feasibility of detecting SL-RNA in these preparations using qPCR and droplet digital PCR. Because the selected SL-RNA target is conserved among *Leishmania* species associated with both VL and CL, the approach was investigated as a potential pan-*Leishmania* detection strategy rather than an assay specific to either VL or CL [22,23]. This study was designed to assess experimental feasibility and not to establish analytical sensitivity, diagnostic accuracy, or clinical utility.

## 2. Materials and Methods

### 2.1. Parasite and Cell Culture

*Leishmania tropica* (MHOM/AF/87/RUPERT), *Leishmania infantum* (MHOM/TR/3/Adana7), transgenic *Leishmania donovani* (MHOM/SD/62/1S-CL2D) constitutively expressing mCherry, and transgenic *Leishmania major* (MHOM/JL/80/Friedlin) constitutively expressing mCherry [42] were maintained as promastigotes at 27 °C in M199 (MP Biomedicals) under standard culture conditions following previously published protocols [43,44]. Depending on the experiment, two to four independent parasite cultures were used, with each culture established and processed separately.

Axenic amastigotes were generated from *L. donovani* log-stage promastigotes and maintained at 37 °C in a humidified 5% CO_2_ atmosphere in a modified RPMI-1640 medium adjusted to pH 5.5, as previously described [43]. 

*Trypanosoma cruzi* (Dm28c) epimastigotes and *Trypanosoma brucei brucei* (427 29:13 WT) procyclic forms were maintained at 27 °C in LIT medium supplemented with fetal bovine serum (FBS) and in SDM-79 medium supplemented with FBS and GlutaMAX, respectively [45,46].

Human THP-1 monocytic cells (ATCC TIB-202) were cultured at 37 °C in a humidified atmosphere containing 5% CO_2_ in RPMI-1640 medium (Corning, Corning, NY, USA) supplemented with 10% FBS and 1% penicillin–streptomycin (Corning). THP-1 monocytes were differentiated into macrophage-like cells by treatment with 150 nM phorbol 12-myristate 13-acetate (PMA) for 24 h prior to infection experiments.

### 2.2. THP-1 Cell Line Infection

Metacyclic promastigotes were isolated from stationary-phase parasite cultures using a Ficoll-400 density gradient as previously described [47]. Differentiated THP-1 macrophages were infected with metacyclic promastigotes at a multiplicity of infection (MOI) of 10:1 parasites per macrophage for 6 h. Following infection, the macrophages were washed five times with phosphate-buffered saline (PBS) to minimize the carryover of extracellular parasites. Immediately after the fifth wash, the macrophage cultures were examined by light microscopy to assess the presence of residual extracellular parasites. No intact extracellular parasites were observed in the fields examined. The macrophages were then incubated in a fresh complete RPMI-1640 medium for an additional 72 h. Three independent infection experiments were performed. Cytospin slides were prepared following the 6 h incubation from each independent infection experiment and stained with a three-step Hematology Quick Stain (VWR) for visual analysis by light microscopy. Infected macrophages (100 total) were examined to calculate the infection rate (% infected) and the parasite indices (# parasites per 100 cells) for each infection sample. The infection rate was calculated as the number of infected macrophages divided by the total number of macrophages examined and multiplied by 100. The infection index was defined as the total number of intracellular parasites counted per 100 macrophages examined. Conditioned culture media were collected from separate infected and uninfected wells for EV enrichment and molecular analyses, consistent with previously established protocols [48]. The experiments were designed to establish qualitative detection of SL-RNA rather than to compare its abundance among sample groups. The conditioning duration and the volume of conditioned medium processed were held constant at 72 h and 5 mL, respectively. No post-collection normalization based on cell number, cell viability, total protein, particle concentration, or recovered RNA was applied. Accordingly, Ct values were interpreted qualitatively and were not used for quantitative comparisons among samples or to infer a relationship between SL-RNA abundance and infection burden.

### 2.3. Mice Infection Model

In this study, 6–8-week-old female wild-type C57Bl/6 mice were bred and housed in the Friemann Life Science Center at the University of Notre Dame. The study was conducted in accordance with national guidelines for animal welfare and was approved by the University of Notre Dame Institutional Animal Care and Use Committee (IACUC) under protocol #24-02-8390. For infections, 1 × 10^5^ *L. major* metacyclic promastigotes in 20 µL of PBS were injected intradermally into the outside surface of the ear as previously described [49], and serum was collected 4–6 weeks post-infection and stored at −20° until use.

### 2.4. Polymer-Based Precipitation of EV-Enriched Preparations

EV-enriched preparations were obtained from PCM, conditioned medium I-MP and U-MP, *T. brucei brucei*, and serum from experimentally infected mice using the Total Exosome Isolation reagent formulated for cell-culture medium or serum, as appropriate (Invitrogen, Carlsbad, CA, USA/Thermo Fisher Scientific, Waltham, MA, USA; catalog Nos. 4478359 and 4478360, respectively), according to the manufacturer’s instructions. All samples were first clarified by centrifugation at 2000× *g* for 30 min to remove cells and cellular debris, and the supernatants were transferred to fresh tubes without disturbing the pellets. For each preparation, 5 mL of conditioned medium or 500 μL of serum was processed, and the recovered pellet was resuspended in 200 μL of 1× PBS. An aliquot of each preparation was stored at −20 °C for short-term storage until particle characterization, whereas the remaining material was used immediately for molecular analysis. Three independent biological collections were processed for each culture-derived sample group, PCM, I-MP, and U-MP, whereas serum samples from individual animals were processed separately. Polymer-based precipitation can co-isolate non-vesicular extracellular material, including protein–RNA complexes and other particles. Therefore, the recovered material is described as an EV-enriched preparation rather than as isolated or purified EVs. NTA was used only to measure particle size and concentration and was not considered sufficient to establish vesicle identity or preparation purity.

### 2.5. Complementary Enrichment of EV-Enriched Preparations by Differential Ultracentrifugation

As a complementary enrichment method, EV-enriched preparations were obtained by differential ultracentrifugation from three independent biological replicates of *L. donovani* PCM maintained under the conditions described above. For each biological replicate, 5 mL of PCM was transferred to a 15-mL conical tube and diluted to a total volume of 14 mL with sterile PBS. The diluted sample was centrifuged at 3000 rpm for 10 min at 4 °C. The supernatant was transferred to a fresh tube under sterile conditions and passed through a 0.40 μm syringe filter. A 10-mL volume of the filtered supernatant was transferred to a glass ultracentrifuge tube and centrifuged at 28,500 rpm for 1 h at 4 °C. The supernatant was carefully removed, leaving approximately 300 µL above the pellet, and the remaining suspension was agitated by gentle shaking and vortexing for 5 s. The pellet was washed by adding 10 mL of sterile PBS and repeating the ultracentrifugation at 28,500 rpm for 1 h at 4 °C. Following removal of the wash supernatant, approximately 300 µL was retained in the tube, and the preparation was resuspended by repeated pipetting followed by gentle vortexing for 3 s to obtain a homogeneous suspension. The resulting EV-enriched preparation was divided into 50 µL aliquots and stored at −80 °C until molecular analysis. Any remaining preparation was reserved for nanoparticle tracking analysis.

This complementary method was used to determine whether SL-RNA remained detectable following an enrichment procedure independent of polymer-based precipitation. Ultracentrifugation alone does not establish vesicle identity or preparation purity; therefore, the recovered material is described as an EV-enriched preparation rather than as purified EVs.

### 2.6. RNase Treatment Assay

To reduce the contribution of unprotected extracellular RNA and assess whether the detected SL-RNA was RNase-protected, EV-enriched preparations derived from PCM, I-MP, U-MP, axenic–amastigote conditioned medium, and ultracentrifugation-derived EV-enriched PCM were treated with RNase A at a final concentration of 100 U/mL (Norgen Biotek Corp., Thorold, ON, Canada). Samples were incubated at 37 °C for 20 min. Three independent biological samples were analyzed for each sample group. Matched untreated aliquots of the PCM, I-MP, and U-MP preparations were processed in parallel as controls. RNase activity was terminated by immediately adding the RNA lysis buffer supplied with the Monarch Total RNA Miniprep Kit (New England Biolabs, Ipswich, MA, USA) and processed directly for column-based RNA extraction. The extracted RNA was analyzed for SL-RNA by reverse transcription-qPCR as described below.

RNase A treatment was performed without detergent-mediated membrane disruption. Therefore, persistence of the SL-RNA signal after RNase treatment was interpreted as evidence of resistance to RNase exposure but not as proof that SL-RNA was enclosed within membrane-bound extracellular vesicles.

### 2.7. Nanoparticle Tracking Analysis (NTA) of EV-Enriched Preparations

To obtain sufficient volume for particle analysis, aliquots of EV-enriched preparations from three independent biological collections within each sample group were pooled before NTA. Pooling of EV-enriched preparations for downstream particle analysis has been described in previous EV studies when sample yield is limited [50]. Consequently, each NTA sample represented a pool of three biological collections rather than three independently analyzed biological replicates. The pooled EV-enriched samples were diluted 10- to 100-fold and analyzed by NTA using a NanoSight LM10 equipped with an sCMOS camera (NanoSight Ltd., Malvern, UK) and a 532 nm laser. The camera level was set to 12, the screen gain was set to 10, and the focus was adjusted manually. For each pooled sample, 500 µL was manually injected into the laser chamber, and five 45 s videos were recorded as technical measurement replicates. The laser chamber was washed with distilled water between samples. Particle size distribution and concentration were analyzed using NanoSight NTA software version 3.1 (NanoSight Ltd.).

### 2.8. RNA Extraction and cDNA Synthesis from EV-Enriched Preparations

Total RNA was extracted from EV-enriched pellets and infected and uninfected mouse serum samples using the Monarch Total RNA Miniprep Kit (New England Biolabs) according to the manufacturer’s instructions. RNA was eluted in nuclease-free water and stored at −80 °C until analysis. RNA concentration and absorbance-based purity were assessed using a NanoDrop spectrophotometer (Thermo Fisher Scientific, Waltham, MA, USA). RNA integrity was not independently evaluated. Complementary DNA (cDNA) was synthesized from 10 µL of purified RNA from each sample using SuperScript III Reverse Transcriptase (Invitrogen) (Thermo Fisher Scientific) according to the manufacturer’s protocol. Because RNA yields from the EV-enriched preparations were low, a fixed RNA volume rather than a normalized RNA mass was used for cDNA synthesis. The resulting cDNA was used immediately for downstream PCR analyses or stored at −20 °C until analysis.

### 2.9. Polymerase Chain Reaction (PCR) and Quantitative PCR (qPCR)

Detection of *Leishmania* SL-RNA was performed using the previously published pan-*Leishmania* SL-RNA primer set [23]. Conventional PCR was performed using GoTaq G2 Green Master Mix (Promega, Madison, WI, USA) according to the manufacturer’s recommendations. Amplified products were analyzed by agarose gel electrophoresis to verify the expected product size before sequencing. qPCR was performed in a total reaction volume of 20 µL using Luna Universal qPCR Master Mix (New England Biolabs). Each reaction contained 1 µL each of the 10 µM forward- and reverse-primer stocks, corresponding to a final concentration of 0.5 µM for each primer. The cycling program comprised 50 °C for 2 min, initial denaturation at 95 °C for 2 min, and 40 cycles of 95 °C for 20 s, 45.7 °C for 30 s, and 68 °C for 30 s. Melt-curve analysis was performed at 95 °C for 15 s, 47 °C for 15 s, and 95 °C for 15 s. For qPCR analysis without RNase treatment, culture-derived sample groups were analyzed using three independent biological replicates, except for the axenic–amastigote preparation, for which one biological replicate was analyzed. A separate qPCR experiment was performed after RNase treatment using three independent biological samples for each RNase-treated sample group, including the axenic–amastigote group, as described above. Each biological sample was evaluated in three technical qPCR replicate reactions. No-template controls and EV-enriched preparations derived from uninfected cells or animals were included as negative controls to monitor contamination and nonspecific amplification.

Because parasite input, recovered RNA mass, and cDNA mass were not standardized across all sample groups, Ct values were interpreted qualitatively as evidence of SL-RNA detection and were not used for quantitative comparisons across species or sample types. No formal analytical limit-of-detection or analytical-sensitivity study was performed.

### 2.10. Droplet Digital PCR (ddPCR)

ddPCR was used as an orthogonal method to confirm *Leishmania* SL-RNA and estimate the target concentration in the analyzed reactions. Reactions were performed in a total volume of 22 µL using the QX200 Droplet Digital PCR (ddPCR) System (Bio-Rad Laboratories, Hercules, CA, USA) according to the manufacturer’s instructions. Each reaction contained 1.1 µL of a mixed forward- and reverse-primer stock (10 µM) and 2.2 µL of cDNA. The reaction mixture was partitioned using the QX200 ddPCR EvaGreen Supermix (Bio-Rad) according to the manufacturer’s instructions. The reaction mix was partitioned into droplets using the QX200 Droplet Generator (Bio-Rad). Thermal cycling was performed under the following conditions: an initial enzyme activation step at 95 °C for 3 min, followed by 40 cycles of 95 °C for 30 s, 49.3 °C for 30 s, and 72 °C for 1 min. Following amplification, reactions were incubated at 4 °C for 5 min and 90 °C for 5 min before being held at 4 °C until analysis. Amplified droplets were analyzed using the QX200 Droplet Reader (Bio-Rad), and data were processed using QuantaSoft Analysis Pro Software (Bio-Rad). Positive and negative droplet populations were identified by applying a fluorescence threshold based on NTCs and negative control samples included in each run. No predefined minimum accepted droplet count was applied. The ddPCR results represented three independent biological samples. Because formal limit-of-detection, analytical-sensitivity, and quantitative-performance studies were not performed, ddPCR results were interpreted as orthogonal confirmation of SL-RNA detection rather than as validation of assay sensitivity or diagnostic performance.

### 2.11. Agarose Gel Electrophoresis and Sequencing Validation

PCR amplicons generated using the *Leishmania* SL-RNA primer set were analyzed by agarose gel electrophoresis to confirm amplification of products of the expected size. To verify amplicon identity, selected PCR products were cloned into the pGEM-T Vector System (Promega) according to the manufacturer’s instructions. Recombinant plasmids were transformed into competent bacterial cells, and putative positive colonies were screened by colony PCR using both SL-RNA primers and M13 forward and reverse primers. Plasmid DNA was extracted from positive colonies, quantified using a NanoDrop spectrophotometer, and re-amplified by PCR to confirm the presence of the target insert. Six confirmed clones, with one clone derived from each of six different biological samples, were submitted for bidirectional Sanger sequencing using M13 forward and reverse primers at the Notre Dame Genomics & Bioinformatics Core Facility, University of Notre Dame, Notre Dame, IN, USA (RRID: SCR_025555). Sequence identity was verified by comparison with published *Leishmania* spliced leader RNA sequences.

## 3. Results

### 3.1. Particle Characterization of EV-Enriched Preparations and RNA Recovery

Pooled EV-enriched preparations obtained from PCM, I- and U-MP CCM, and mouse serum were analyzed by NTA to determine particle size and concentration (Table 1). Most preparations had mean particle diameters below approximately 200 nm. NTA characterized the particles present in the preparations but did not establish vesicle identity or preparation purity. Corresponding particle-size distribution plots are provided in Supplementary Figure S1. Total RNA was recovered from the EV-enriched preparations, and the measured RNA concentrations are reported in Supplementary Table S1.

### 3.2. Standard Curve Assessment of the SL-RNA qPCR Assay

**Figure 1:**
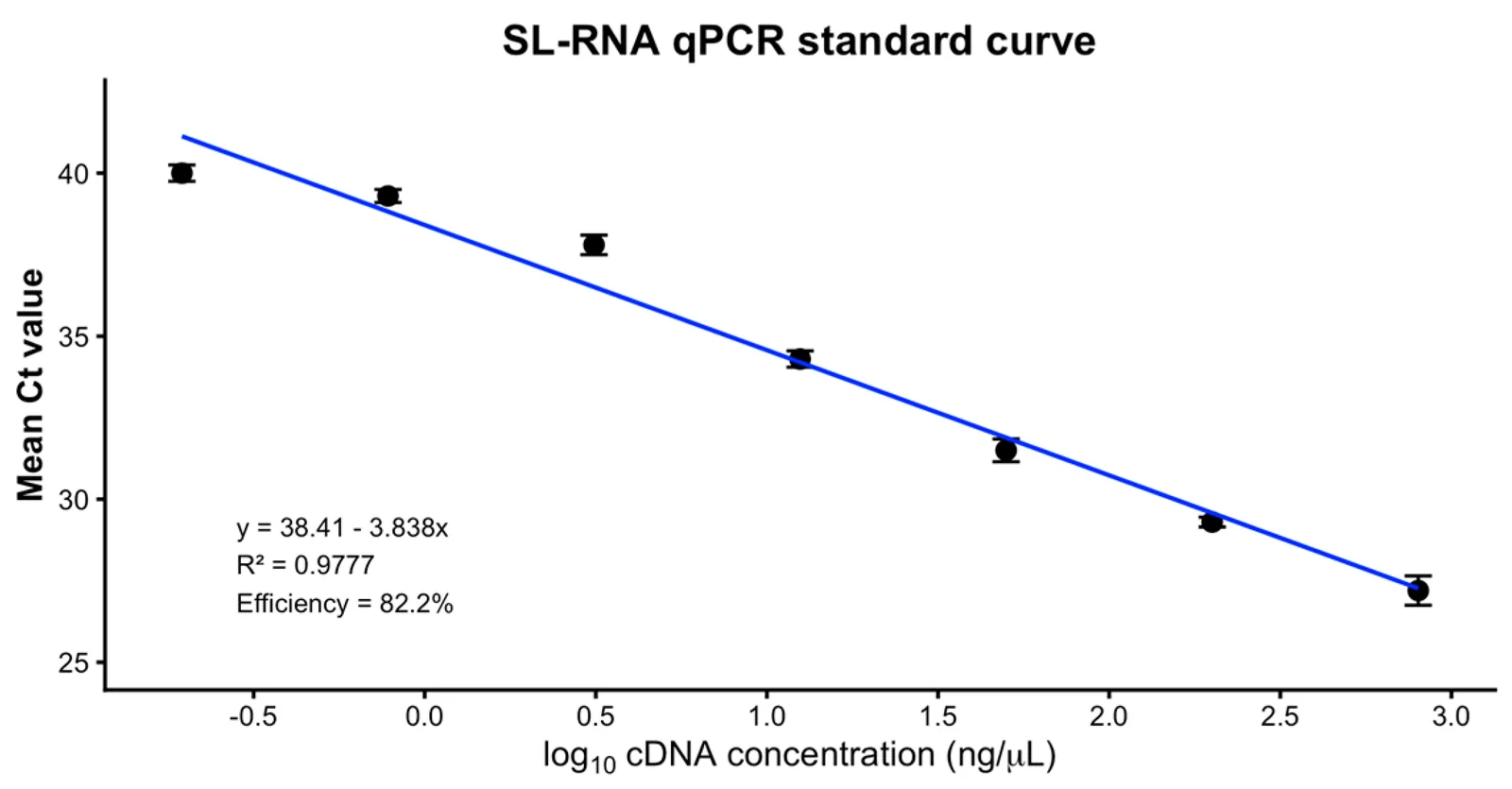
Standard curve assessment of the *L. donovani* SL-RNA qPCR assay. Seven fourfold serial dilutions of *L. donovani* cDNA, ranging from approximately 0.20 to 800 ng/µL, were analyzed by qPCR. Mean Ct values were plotted against the log_10_-transformed cDNA concentration. Black circles represent mean Ct values, error bars denote standard deviations from three technical replicates, and the blue line represents the least-squares linear regression fit.

A standard curve was generated using seven fourfold serial dilutions of *Leishmania donovani* cDNA, covering concentrations from approximately 0.20 to 800 ng/µL. Mean Ct values decreased progressively with increasing cDNA concentration, from approximately 40.0 at the lowest input concentration to 27.2 at the highest concentration (Figure 1). Linear regression of mean Ct against log_10_-transformed cDNA concentration produced the equation y = 38.41 − 3.838×, with a coefficient of determination of R^2^ = 0.9777. The regression slope corresponded to an amplification efficiency of 82.2%, indicating suboptimal efficiency under the tested conditions. The standard curve demonstrated concentration-dependent amplification across the approximately 3.6-log_10_ tested input range. It was not used to establish quantitative assay performance, and no formal limit of detection or analytical sensitivity was determined. 

### 3.3. Detection of Leishmania SL-RNA in EV-Enriched Preparations Across Experimental Models

#### 3.3.1. SL-RNA Detection in EV-Enriched Preparations from Four Leishmania Species and Axenic Amastigotes

EV-enriched preparations obtained from PCM were analyzed by RT-qPCR. SL-RNA was detected in RNA extracted from EV-enriched preparations from all four tested *Leishmania* species: *L. donovani* (Ld), *L. major* (Lm), *L. infantum* (Li), and *L. tropica* (Lt). For each promastigote-derived sample group, three independent biological collections were analyzed, with each biological sample evaluated in three technical qPCR reactions. The technical reactions were averaged for each biological sample before summary analysis.

**Figure 2:**
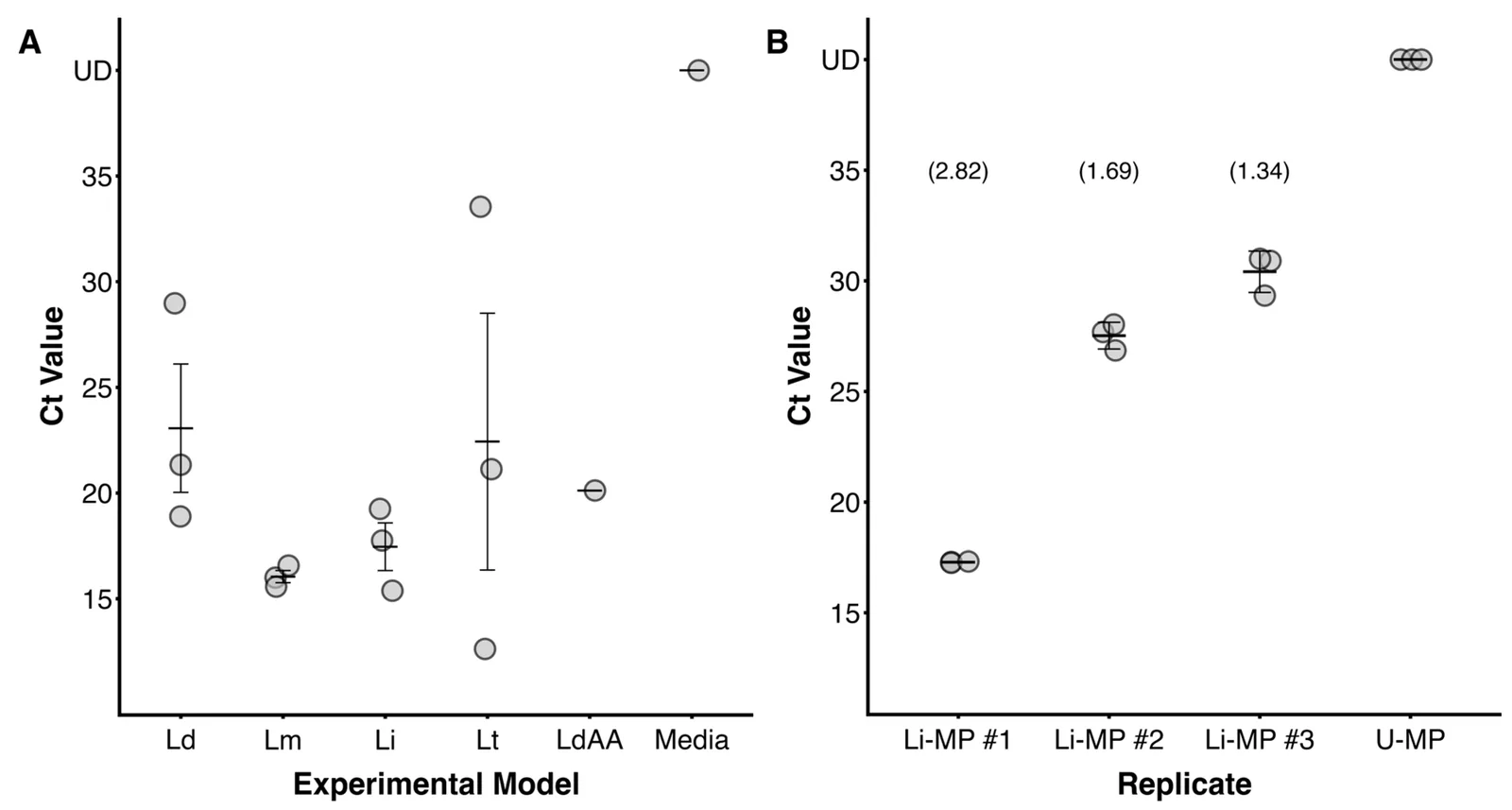
qPCR detection of SL-RNA in EV-enriched preparations from multiple *Leishmania* experimental models. (**A**) EV-enriched preparations from PCM of *L. donovani* (Ld), *L. major* (Lm), *L. infantum* (Li), *L. tropica* (Lt) promastigotes, and *L. donovani* axenic amastigotes (LdAAs) were analyzed for SL-RNA. Each point represents the mean of three technical replicates for one biological sample. Horizontal lines indicate means of three biological replicates ± SE. The LdAA result represents one biological sample. SL-RNA was detected in all parasite-derived preparations but not in the media-derived sample. (**B**) EV-enriched preparations from uninfected THP-1 macrophages (U-MP) and *L. infantum*-infected THP-1 macrophage (Li-MP)-conditioned media were analyzed for SL-RNA. Points represent technical replicates of three biological samples; horizontal lines indicate means ± SD. SL-RNA was detected in all Li-MP-derived preparations, whereas amplification was not detected in U-MP preparations. The mean intracellular parasite burden is presented in parentheses above each biological sample. UD, no amplification detected within 40 cycles.

SL-RNA was detected in all three biological collections from each promastigote group. The mean Ct values were 23.07 ± 5.28 for Ld, 16.05 ± 0.50 for Lm, 17.46 ± 1.94 for Li, and 22.43 ± 10.46 for Lt (Figure 2A). Because parasite input, recovered RNA quantity, and cDNA amounts were not standardized across species, these Ct values were interpreted qualitatively and were not compared quantitatively among species. SL-RNA was also detected in one biological preparation obtained from *L. donovani* axenic–amastigote (LdAA) conditioned medium (Ct = 20.12; n = 1), providing preliminary evidence of detection in this alternative developmental stage. Corresponding amplification plots are presented in Supplementary Figure S2.

Target-consistent amplification produced a single melt peak corresponding to the validated SL-RNA amplicon. No amplification was detected within 40 cycles in the fresh-M199 process control or no-template controls.

#### 3.3.2. Detection of SL-RNA in EV-Enriched Preparation from Leishmania-Infected Macrophages

Differentiated THP-1 macrophages were infected with *L. infantum* metacyclic promastigotes, and EV-enriched preparations obtained from I-MP and -MP conditioned medium were analyzed by RT-qPCR. Immediately after the fifth post-infection wash, no intact extracellular parasites were observed by light microscopy in the examined fields. Infection measurements obtained after the 6 h infection period showed infection rates of 68%, 48%, and 42% for independent experiments 1, 2, and 3, respectively. The corresponding mean intracellular parasite burdens were 2.82, 1.69, and 1.34 parasites per infected macrophage.

SL-RNA was detected in EV-enriched preparations from all three independent I-MP experiments. Each biological sample was analyzed in three technical qPCR reactions, yielding mean Ct values of 17.29 ± 0.03, 27.52 ± 0.60, and 30.41 ± 0.93 for experiments 1, 2, and 3, respectively (Figure 2B). Ct variation was interpreted qualitatively and was not used to infer a quantitative relationship between SL-RNA abundance and infection burden because only three independent experiments were analyzed, and the samples were not normalized for quantitative comparison. No amplification was detected within 40 cycles in the U-MP preparations. Representative amplification plots are provided in Supplementary Figure S3.

#### 3.3.3. Detection of SL-RNA in Serum EV-Enriched Preparations from Leishmania-Infected Mice

**Figure 3:**
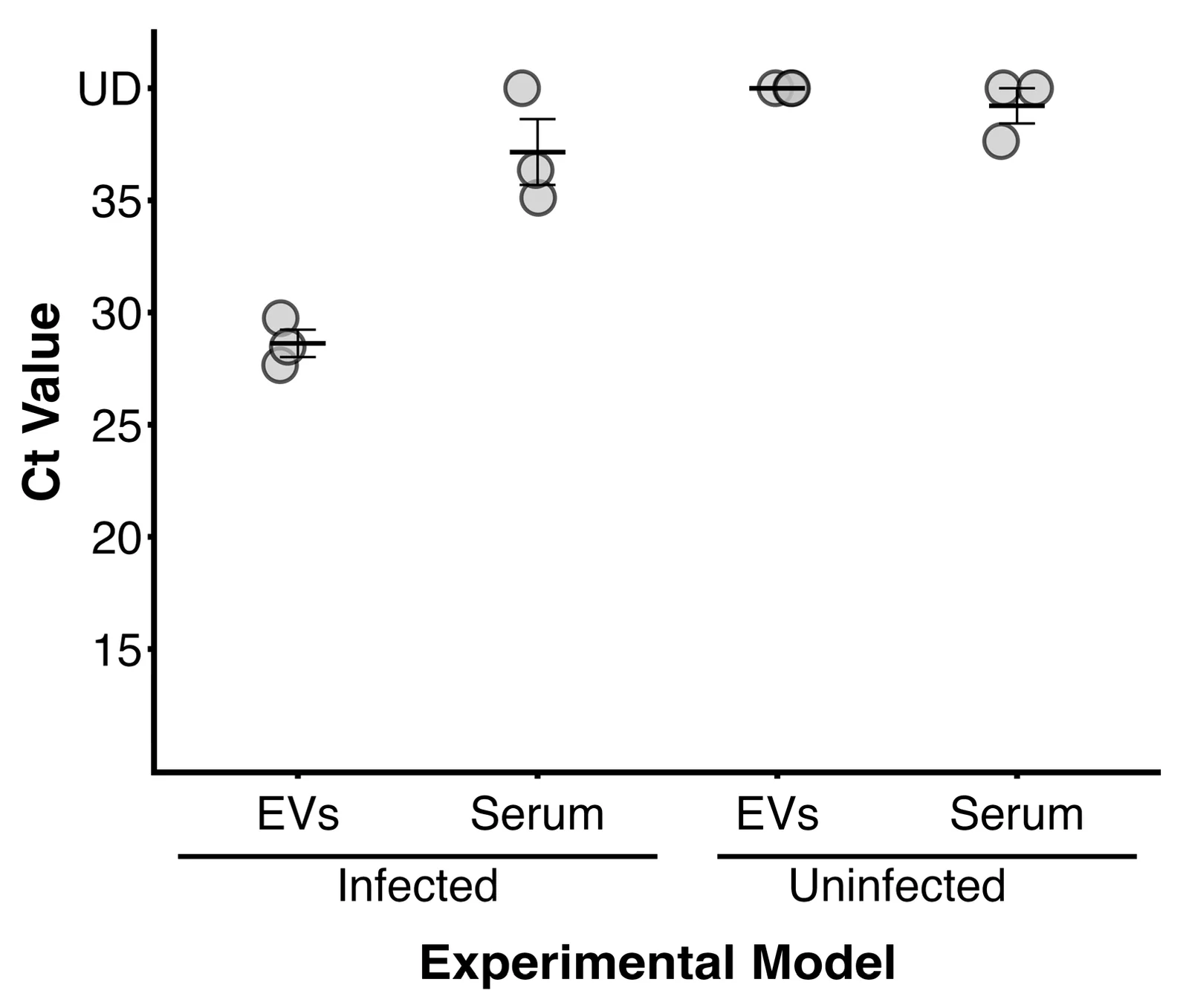
RT-qPCR detection of SL-RNA in serum and serum-derived EV-enriched preparations from *Leishmania*-infected mice. RNA extracted directly from serum and from matched serum-derived EV-enriched preparations of three *L. major*-infected and three uninfected C57BL/6 mice was analyzed by RT-qPCR. Points represent individual mice and show mean Ct values from three technical replicates. Horizontal bars indicate mean ± SD. UD, undetermined amplification.

RNA extracted directly from serum and from corresponding serum-derived EV-enriched preparations of three *L. major*-infected and three uninfected C57BL/6 mice was analyzed by qPCR. Each sample was analyzed in three technical replicates. The EV-enriched preparations from infected mice showed lower Ct values and consistent amplification compared to the matched serum samples (Figure 3), whereas all uninfected controls remained undetermined. Furthermore, SL-RNA was detected in serum-derived EV-enriched preparations from an additional eight infected mice, with mean Ct values ranging from 28.82 to 33.36 and a median of 31.19 (Supplementary Figure S4A,B).

#### 3.3.4. Detection of RNase-Resistant SL-RNA in Polymer-Precipitated and Ultracentrifugation-Derived EV-Enriched Preparations

To assess whether the SL-RNA signal was protected from extracellular RNase exposure, EV-enriched preparations obtained by polymer-based precipitation and differential ultracentrifugation were treated with RNase A. RNase treatment reduced the amplification signal in the positive sample groups: polymer-precipitated preparations from *L. donovani* PCM, *L. donovani* axenic–amastigote conditioned medium, and I-MP, as well as differential-ultracentrifugation-derived *L. donovani* PCM. However, SL-RNA remained detectable and distinguishable from the uninfected-macrophage, fresh-medium, and no-template controls (Supplementary Figure S5). These findings demonstrated resistance to RNase exposure under the tested conditions and confirmed SL-RNA detection from EV-enriched LdAA conditioned medium.

### 3.4. Orthogonal Confirmation of SL-RNA Detection by ddPCR

**Figure 4:**
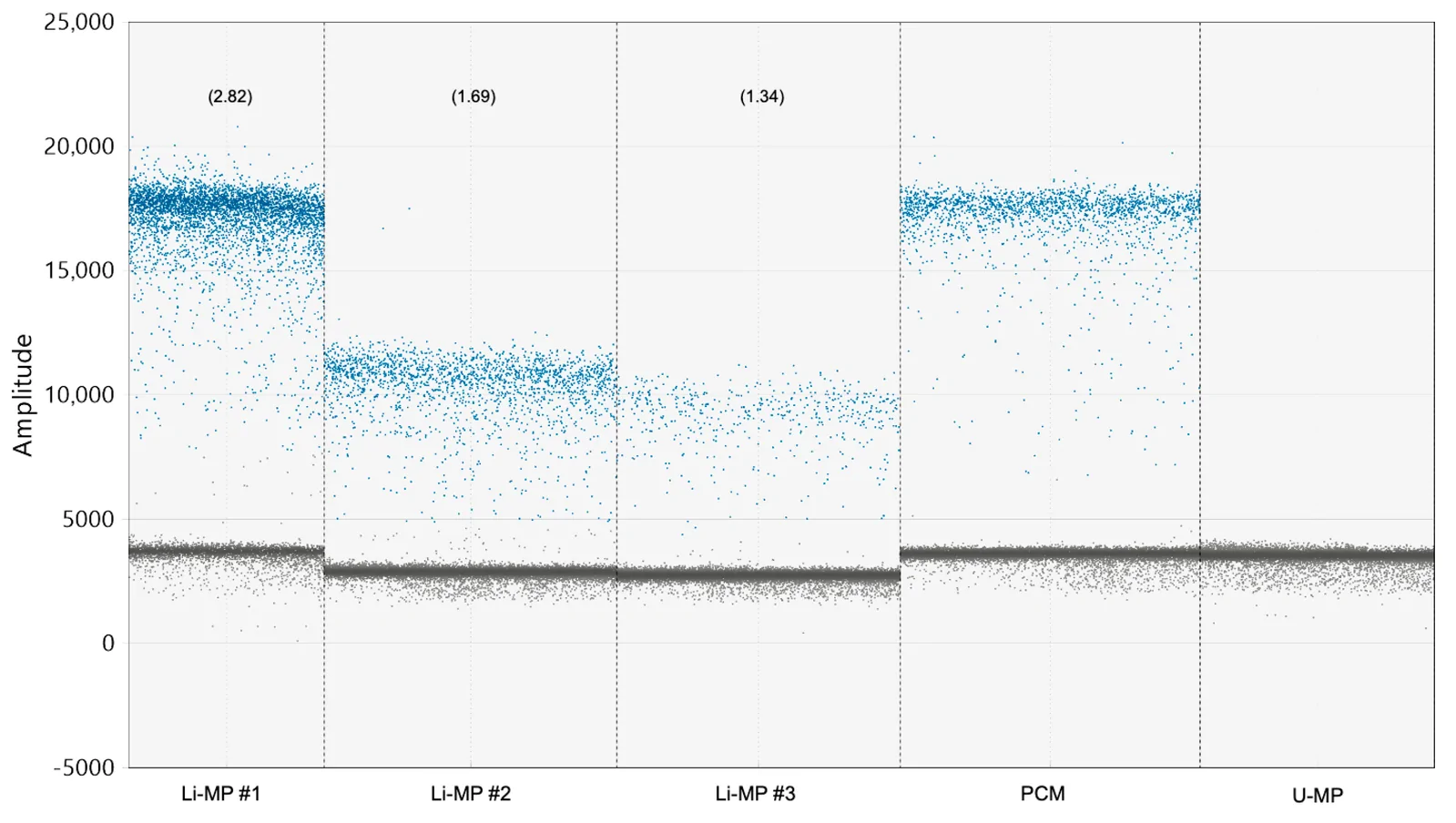
Orthogonal confirmation of SL-RNA detection in EV-enriched preparations by ddPCR. Droplet-amplitude plots of EV-enriched preparations from Li-MP #1–3, PCM, and U-MP CCM were analyzed for SL-RNA by ddPCR. Positive droplets were detected in the three independent I-MP samples and the PCM positive control, whereas no positive droplets were detected in the U-MP preparation. The mean intracellular parasite burden is presented in parentheses above each I-MP sample.

RNA derived from I-MP EV-enriched preparations was analyzed by ddPCR. A PCM EV-enriched sample was included as a positive control, while RNA from U-MP preparation and an NTC served as negative controls. Positive droplets corresponding to SL-RNA were detected in all three I-MP samples and the PCM control. In contrast, no positive droplets were observed in the U-MP sample (Figure 4). These results were interpreted as orthogonal confirmation of SL-RNA detection rather than as validation of analytical sensitivity or quantitative performance.

### 3.5. Sequence Confirmation of the Amplified SL-RNA

The identity of the amplified SL target was confirmed by Sanger sequencing. Six clones, with one clone derived from each of six different biological samples, contained the expected 39 nt *Leishmania* SL sequence. Representative bidirectional reads showing 100% identity to the reference sequence are shown in Supplementary Figure S6. The complete nucleotide sequences for all six clones are provided in Supplementary Figure S7.

### 3.6. Cross-Reactivity Assessment of the Leishmania SL-RNA Assay

**Figure 5:**
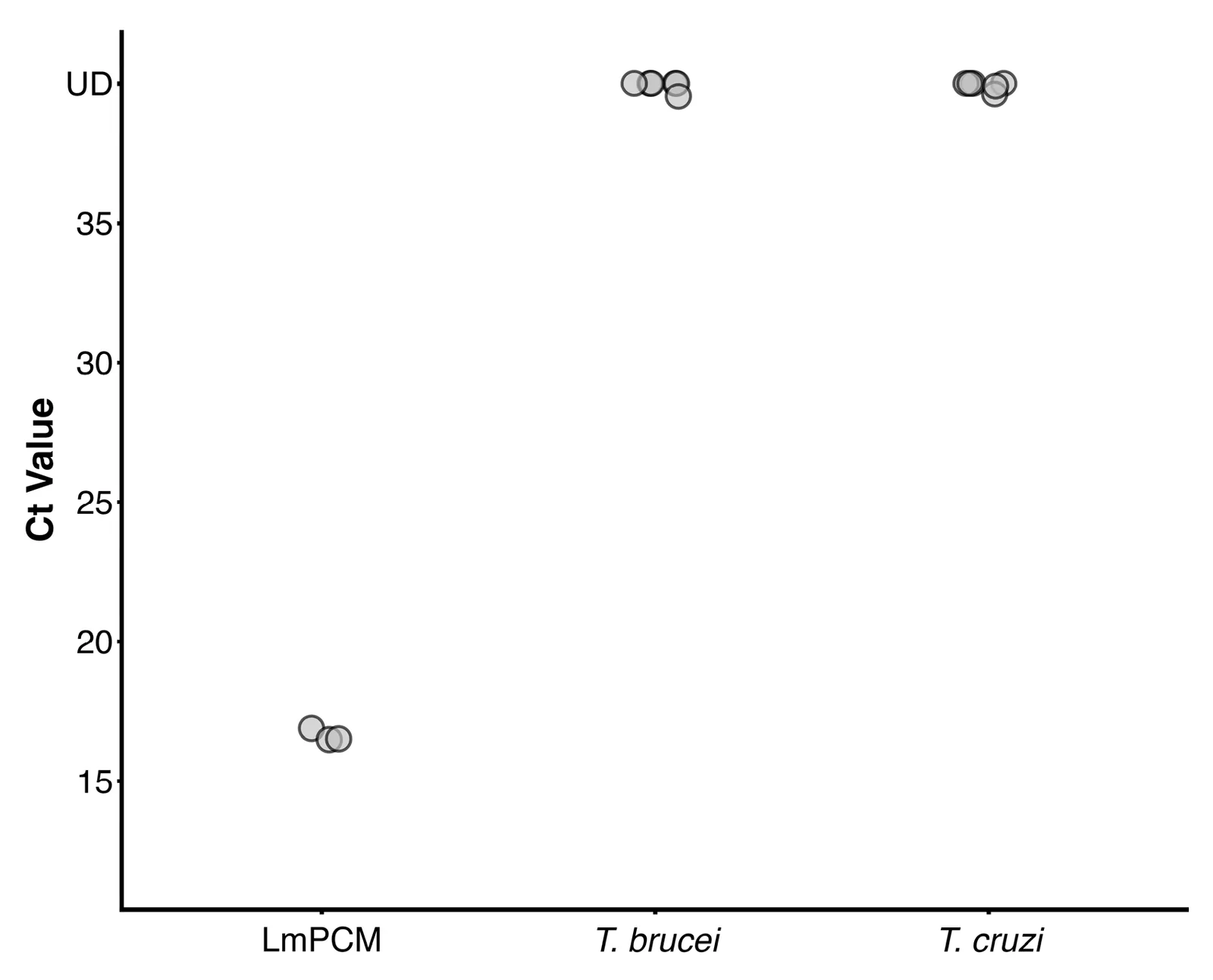
Cross-reactivity assessment of SL-RNA using non-target kinetoplasts.

Cross-reactivity was assessed using RNA extracted from EV-enriched preparations from *L. major* PCM and the closely related non-target trypanosomatids, *T. brucei brucei* (Tbb) and *T. cruzi* (Tc). Two independent biological samples from each non-target species were analyzed, with three technical replicates per sample. The *L. major* PCM sample produced strong and consistent amplification in all three replicates, with a mean Ct of 16.62 ± 0.23 (Figure 5). In contrast, the replicates of Tbb and Tc produced Ct values more than 22 cycles later than the mean *L. major* signal. Because a predefined positivity threshold was not established, these limited data were not considered sufficient to establish analytical specificity. RT-qPCR amplification plots are presented in Supplementary Figure S3.

EV-enriched preparations from Lm-PCM, Tbb-PCM, and Tc-PCM were analyzed for SL-RNA by qPCR. Points represent technical replicates of Lm-PCM (n = 1 biological sample), Tbb-PCM (n = 2 biological samples), and Tc-PCM (n = 2 biological samples). SL-RNA was detected in Lm-PCM, whereas it was not detected in Tbb-CCM and Tc-CCM.

## 4. Discussion

This proof-of-concept study demonstrated that *Leishmania*-derived SL-RNA can be detected in EV-enriched preparations from PCM, I-MP, and serum from experimentally infected mice. Detection across four *Leishmania* species and different parasite stages, together with the absence of target-consistent amplification in corresponding controls and sequence confirmation from six biological samples, supports the experimental feasibility of this approach to develop a reliable biomarker.

Previous studies identified SL-RNA as a sensitive pan-*Leishmania* target in clinical samples, experimentally infected animals, vectors, and reservoir hosts [19,23]. Its abundance and association with parasite transcription make it a potentially useful marker of active infection. Detection in species associated with visceral and cutaneous leishmaniasis supports further evaluation as a pan-*Leishmania* approach, although the species panel was not expansive. The Ct values in this study cannot be compared across species, stages, or sample types due to unstandardized parasite input, particle recovery, RNA yield, and cDNA quantity. Instead, these results simply confirm that SL-RNA is detectable in EV-enriched samples across multiple model systems.

SL-RNA was detected in all three I-MP preparations and all infected mouse serum preparations, but not in their respective controls. This result is consistent with evidence that EV-enriched preparations carry parasite- and host-derived molecules with potential diagnostic relevance [33,34,35,36,37,38,51]. However, although no intact extracellular parasites were observed after the fifth macrophage wash, microscopy cannot exclude residual parasite-derived material, and the detected RNA cannot be attributed exclusively to secretion by infected host cells. The macrophage Ct variation also cannot be related quantitatively to infection burden because only three independent experiments were performed, and the samples were not normalized for quantitative comparison. Likewise, we did not assess lesion size or parasite burden in the infected mouse samples. Although these findings only represent a proof-of-concept for SL-RNA detection in biological fluids, they underscore the diagnostic potential of targeting SL-RNA within EV-enriched samples.

The physical location of the detected SL-RNA remains unresolved. Polymer precipitation may co-isolate non-vesicular particles and RNA–protein complexes, while NTA establishes particle size and concentration but not vesicle identity and purity [26]. Detection after differential ultracentrifugation demonstrated that SL-RNA recovery was not restricted to the precipitation method. SL-RNA also remained detectable after RNase A treatment, although the signal was reduced, indicating resistance to RNase exposure. Because RNase-plus-detergent treatment was not performed, this finding does not prove that SL-RNA was enclosed within membrane-bound vesicles. Previous *L. infantum* studies combined ultracentrifugation with transmission electron microscopy, NTA or dynamic light scattering, density measurements, and protein analysis [51,52]. Similar morphological and marker-based characterization, together with RNase treatment with and without membrane disruption, is required to confirm SL-RNA as EV cargo.

The RT-qPCR assay demonstrated concentration-dependent amplification, although its efficiency was suboptimal (82.2%) and formal limits of detection and quantification were not established; therefore, Ct values support qualitative detection only. ddPCR provided orthogonal confirmation, but its advantage over qPCR remains to be evaluated. Although the pan-*Leishmania* SL-RNA assay was previously reported to be specific [23], late amplification occurred in the Tbb and Tc samples, preventing analytical specificity from being established in this study. A broader cross-reactivity panel and predefined positivity threshold are therefore required.

Serum-based detection could reduce reliance on invasive tissue sampling. In the mouse samples, EV-enriched serum preparations produced a stronger RT-qPCR signal than RNA extracted directly from serum, which suggests that enrichment may improve target recovery. However, this preliminary comparison does not establish improved analytical or diagnostic sensitivity and requires confirmation in a larger, standardized sample set. The current multistep workflow also requires specialized equipment and trained personnel. In low-resource settings, initial implementation may therefore be more feasible in regional molecular laboratories. Broader use would require simplified sample preparation, stable reagents, and portable or isothermal amplification platforms.

The main limitations of this study were incomplete EV characterization, low and variable RNA yield, lack of an independent RNA-integrity assessment and formal analytical validation, a limited cross-reactivity panel, and the absence of human samples. Clinical studies should evaluate confirmed visceral and cutaneous leishmaniasis alongside appropriate healthy and disease controls, compare performance with established diagnostic methods, and assess relationships with parasite burden, disease severity, and treatment response. Overall, the findings support proof-of-concept detection of RNase-resistant SL-RNA in EV-enriched experimental preparations, but further analytical and clinical validation is required before it can be considered a minimally invasive diagnostic biomarker.

## 5. Conclusions

This proof-of-concept study demonstrates that *Leishmania* SL-RNA can be detected in EV-enriched preparations from experimental culture, infected-cell, and mouse samples, with ddPCR providing orthogonal confirmation and sequencing confirming amplicon identity. Because vesicular localization, analytical performance, and diagnostic accuracy were not established, these findings do not validate SL-RNA as a clinical biomarker. Further analytical evaluation and studies using well-characterized human samples are required to determine its diagnostic and treatment-monitoring utility.

## Figures and Tables

**Table 1:** NTA measurements of pooled EV-enriched preparations from PCM, I- and U-MP CCM, mouse serum, and negative process controls.

Sample Preparation	Mean Particle Diameter ± SE (nm)	Particle Concentration ± SE (Particles/mL)
*L. donovani* promastigote	231.8 ± 2.0	(2.06 ± 0.16) × 10^9^
*L. major* promastigote	166.8 ± 2.1	(6.37 ± 0.36) × 10^9^
*L. tropica* promastigote	163.0 ± 8.3	(6.62 ± 0.53) × 10^8^
*L. infantum* promastigotes	189.7 ± 4.3	(4.65 ± 0.39) × 10^9^
*L. donovani* axenic amastigote	165.3 ± 1.6	(1.93 ± 0.04) × 10^9^
*T. brucei* procyclic	167.1 ± 2.8	(1.11 ± 0.04) × 10^9^
*T. cruzi* epimastigotes	182.3 ± 4.9	(4.09 ± 0.08) × 10^9^
*L. infantum*-infected THP-1 MP	177.3 ± 3.2	(2.10 ± 0.11) × 10^9^
Uninfected THP-1 MP	172.9 ± 4.6	(2.04 ± 0.19) × 10^9^
*L. major*-infected mouse serum	251.4 ± 10.3	(2.39 ± 0.06) × 10^9^
Uninfected mouse serum	200.2 ± 2.7	(2.42 ± 0.31) × 10^9^
Freshly conditioned medium	ND	2.87 × 10^6^
PBS process control	ND	1.43 × 10^6^ ^1^

^1^ Values are presented as the mean ± standard error of five technical NTA measurements performed on each pooled preparation. Each pooled culture-derived preparation contained material from three independent biological collections. Particle concentrations were corrected for the dilution applied before NTA. ND, not determined.

## Data Availability

The raw data supporting the conclusions of this manuscript will be made available by the author upon request.

## Supplementary Materials

The following supporting information can be downloaded at https://www.mdpi.com/article/10.3390/tropicalmed11100281/s1, Figure S1: Particle-size distribution profiles of EV-enriched preparations measured by NTA; Table S1: RNA concentrations recovered from EV-enriched preparations derived from parasite cultures, THP-1 macrophages, and mouse serum; Figure S2: qPCR amplification of SL-RNA in EV-enriched preparations; Figure S3: Cross-reactivity assessment of SL-RNA amplification in non-target kinetoplasts; Figure S4A: qPCR amplification of SL-RNA in mouse serum EV-enriched preparations; Figure S4B: RT-qPCR detection of SL-RNA in serum-derived EV-enriched preparations; Figure S5: RT-qPCR detection of RNase-resistant SL-RNA in EV-enriched preparations; Figure S6. Sequence confirmation of the amplified Leishmania spliced-leader target. File S1: Sanger sequencing reads and available chromatograms.

## Abbreviations

The following abbreviations are used in this manuscript:

SL-RNA	Spliced Leader RNA
EVs	Extracellular Vesicles
ddPCR	Droplet Digital PCR
NTA	Nanoparticle Tracking Analysis
qPCR	Quantitative PCR
NTC	No-Template Control
CCM	Cell-Conditioned Medium
U-MPs	Uninfected Macrophages
I-MPs	Infected Macrophages
PCM	Parasite-Conditioned Medium
