# Supplementary material for: Extracellular Vesicle-Derived Spliced Leader RNA as a Minimally Invasive Molecular Biomarker for Leishmaniasis Detection
Source: Trop Med Infect Dis. 2026 Oct 3;11(10):281. doi: 10.3390/tropicalmed11100281 (PMC13709751; doi:10.3390/tropicalmed11100281)
Supplement: Supplementary file 1 [file tropicalmed-11-00281-s001.zip › tropicalmed-4505493 supplementary information.pdf]

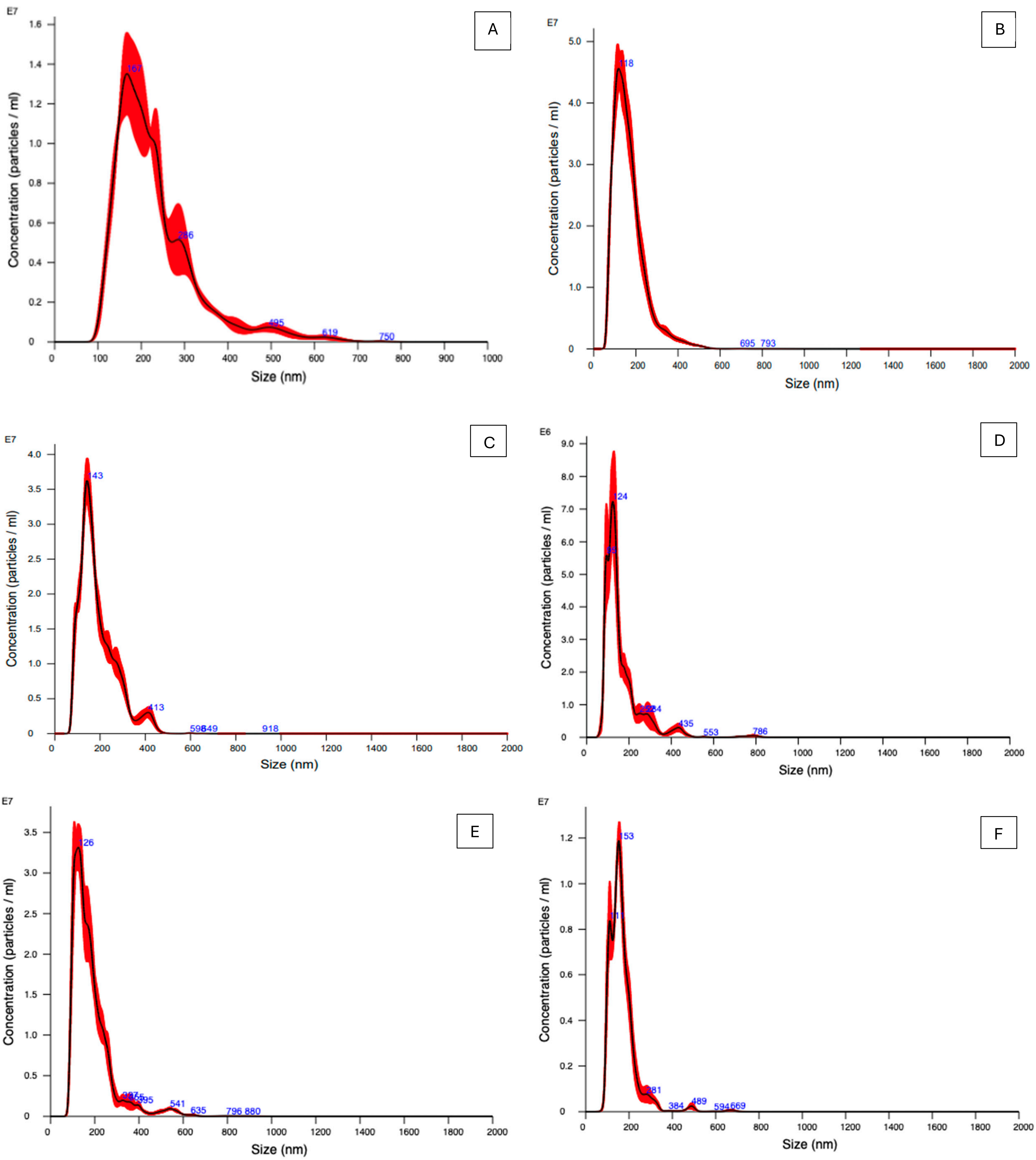

A
E7
Concentration (particles / ml)
Size (nm)
167
286
495
619
750
B
E7
118
695
793
C
E7
143
413
918
D
E6
124
435
553
786
E
E7
126
541
635
796
880
F
E7
153
281
384
489

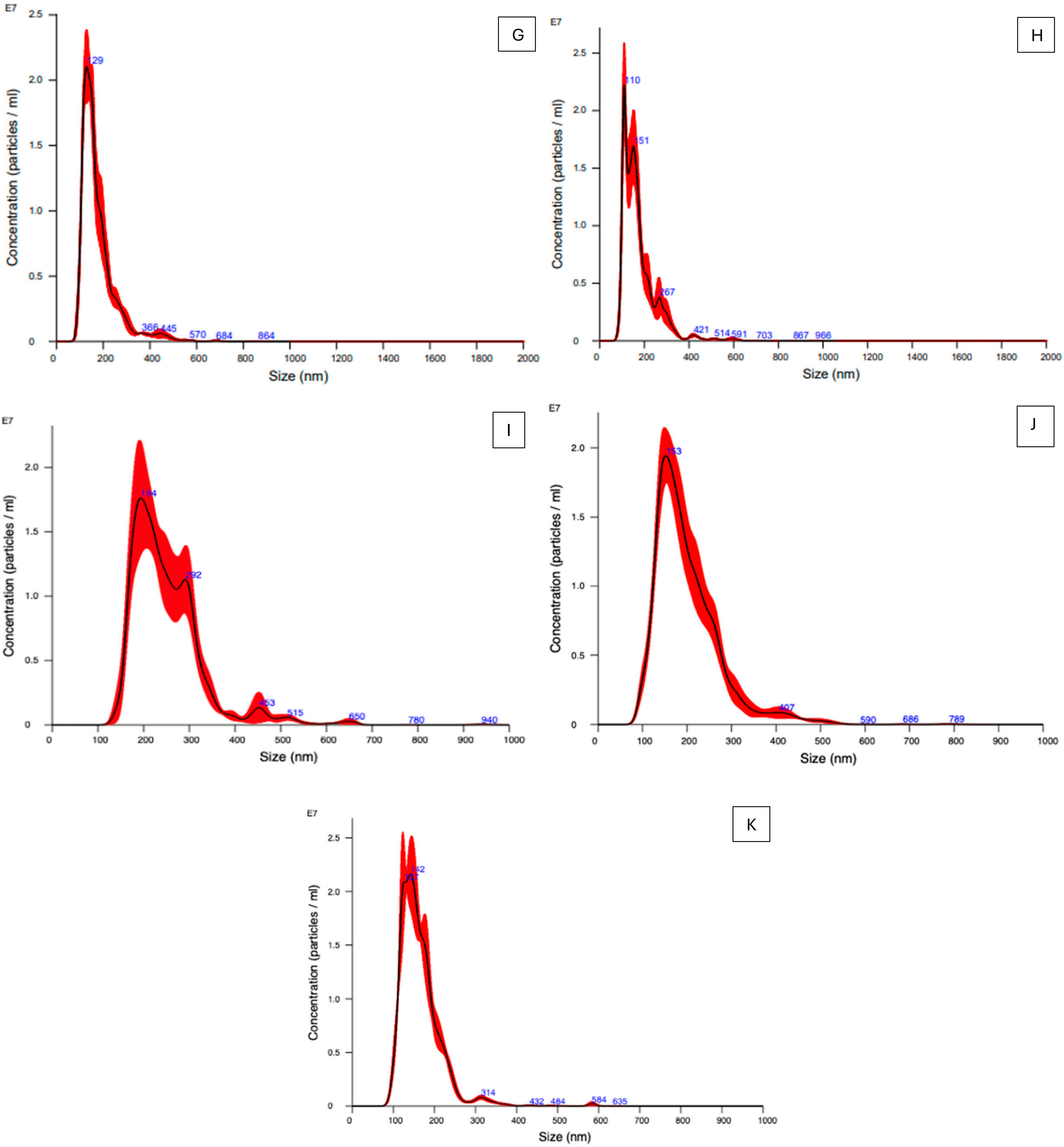
G
E7
Concentration (particles / ml)
Size (nm)
129
366
445
570
684
864
H
E7
Concentration (particles / ml)
Size (nm)
110
151
267
421
514
591
703
867
966
I
E7
Concentration (particles / ml)
Size (nm)
194
292
453
515
650
780
940
J
E7
Concentration (particles / ml)
Size (nm)
153
407
590
686
789
K
E7
Concentration (particles / ml)
Size (nm)
142
314
432
484
584
635

Supplementary Figure S1. Particle-size distribution profiles of EV-enriched preparations measured by NTA.

Representative averaged NTA particle-size distribution profiles of pooled EV-enriched preparations obtained from *Leishmania donovani* (A), *L. major* (B), *L. infantum* (C), *and L. tropica* (D) promastigotes, *Trypanosoma. cruzi* epimastigotes (E); *Trypanosoma brucei brucei* procyclic forms (F); *L. infantum*-infected and uninfected THP-1 macrophage-conditioned media (G and H, respectively); and *L. major*-infected and uninfected mouse serum (I and J, respectively); *L. donovani* axenic amastigotes (K). Profiles represent merged technical NTA measurements for each pooled preparation. Shaded regions or error bars indicate ±1 standard error of the mean, as generated by the NanoSight NTA software.

Table S1. RNA concentrations recovered from EV-enriched preparations derived from parasite cultures, THP-1 macrophages, and mouse serum.

Sample preparation	RNA concentration (ng/µL)
L.donavani promastigote	0.92 ± 0.73
L.major promastigote	1.56 ± 0.11
L.tropica promastigote	1.26 ± 0.18
L.infantum promastigotes	1.49 ± 0.42
L.donavani axenic amastigote	1.40
T. brucei brucei procyclic	1.57 ± 0.46
T.cruzi epimastigotes	1.34 ± 0.54
L.infantum-infected THP-1 MP	5.89 ± 0.99
Uninfected THP-1 MP	5.60
L.major-infected mouse serum	7.32
Uninfected mouse serum	5.89 [1]

[1] RNA concentrations were measured using a NanoDrop spectrophotometer. Values from preparations with multiple measurements are presented as mean ± SD preparations with a single measurement are reported without an error term. All recovered RNA samples were used for cDNA synthesis and subsequent qPCR and ddPCR analyses.

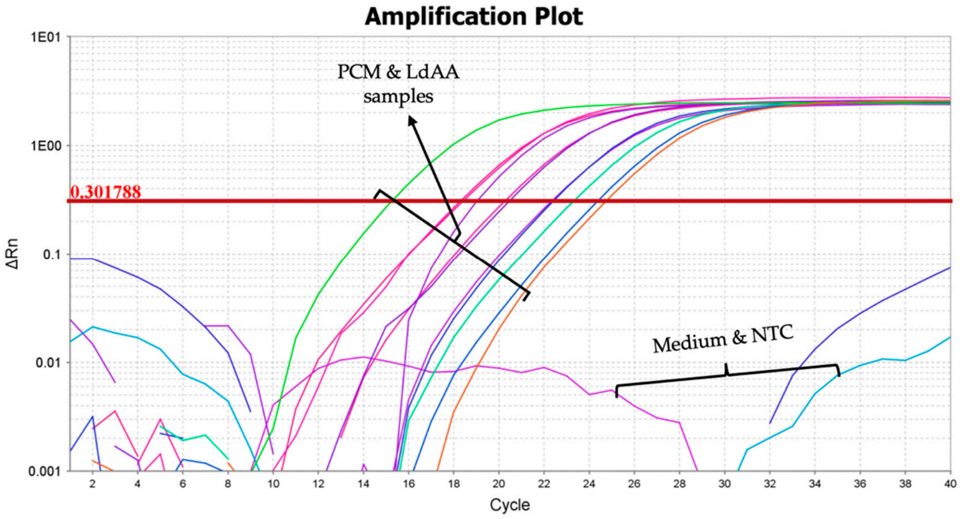

Figure S2. qPCR amplification of SL-RNA in EV-enriched preparations

SL-RNA amplification was detected in EV-enriched preparations from four *Leishmania* species (PCM) and *L. donovani* axenic amastigotes (LdAA). The medium-derived negative control and NTC did not cross the amplification threshold.

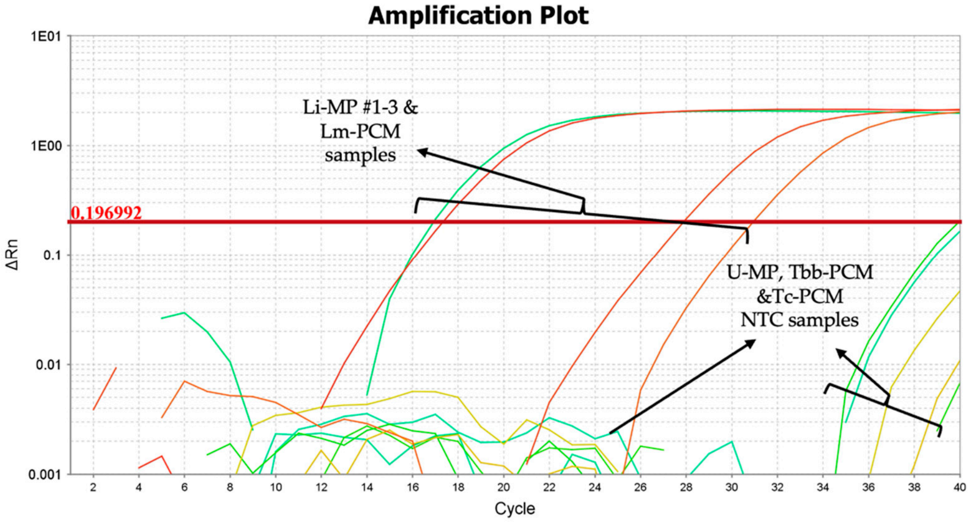

Figure S3. Cross-reactivity assessment of SL-RNA amplification in non-target kinetoplasts.

SL-RNA amplification was detected in EV-enriched preparations from *L. infantum*-infected macrophages (Li-MP #1-3) and *L.major* PCM (Lm-PCM). Uninfected macrophage (U-MP), *T. brucei brucei* (Tbb-PCM), *T. cruzi* (Tc-PCM), and NTC samples did not cross the amplification threshold.

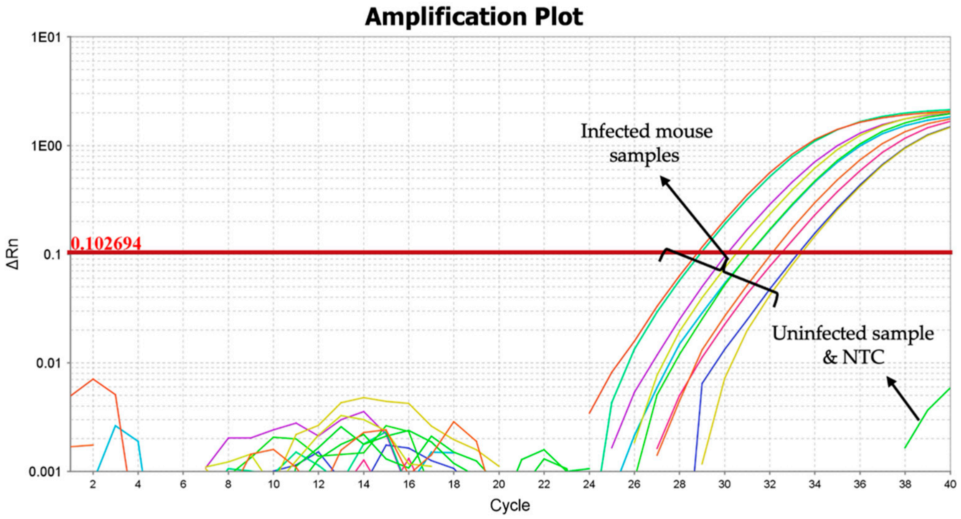

Figure S4. (A). qPCR amplification of SL-RNA in mouse serum EV-enriched preparations. SL-RNA amplification was detected in EV-enriched preparations from *L. major*-infected mouse samples, whereas the uninfected sample and NTC did not cross the amplification threshold.

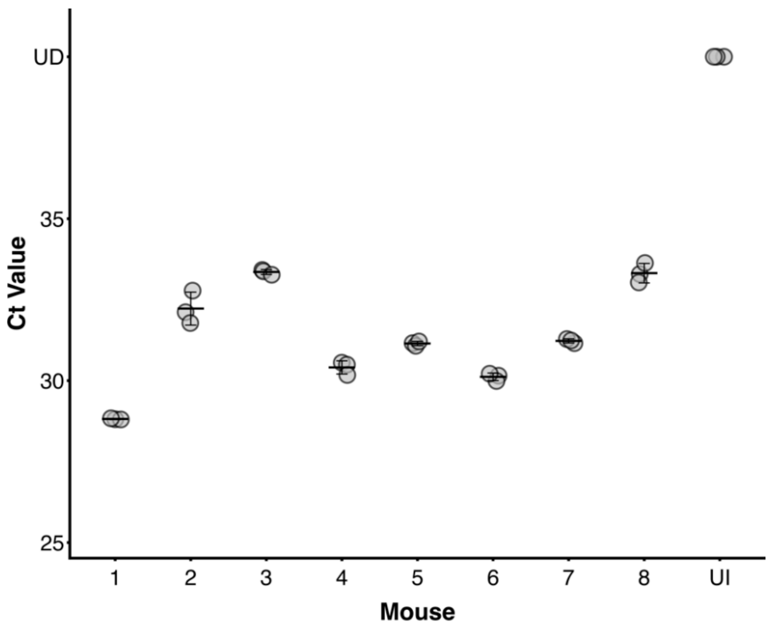

Figure S4. (B) RT-qPCR detection of SL-RNA in serum-derived EV-enriched preparations.

EV-enriched preparations from the serum of eight *L. major*-infected C57Bl/6 mice and one uninfected control mouse were analyzed for SL-RNA. Points represent means of three technical replicates, horizontal lines indicate means of three biological replicates ± SD. SL-RNA was detected in all eight infected mice derived EV preparations compared to no detection in the uninfected mouse (UI) control.

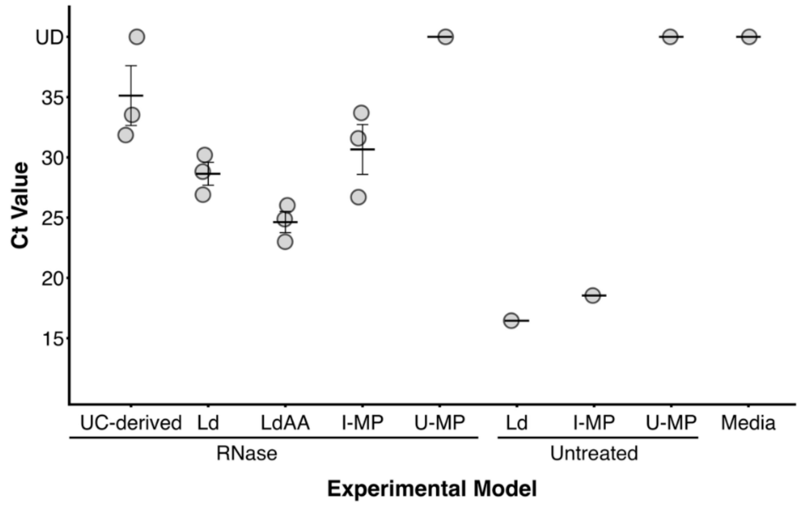

Figure S5. RT-qPCR detection of RNase-resistant SL-RNA in EV-enriched preparations.

Polymer-precipitated preparations from *L. donovani* PCM (Ld), axenic-amastigote-conditioned medium (LdAA), and I-MP, as well as differential-ultracentrifugation-derived *L. donovani* PCM (Ld) preparations, were treated with RNase A. Although RNase treatment reduced amplification, SL-RNA remained detectable and distinguishable from uninfected-macrophage, fresh-medium, and no-template controls.

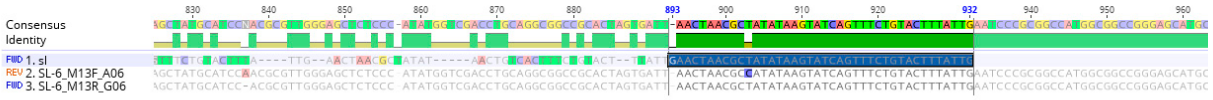

Figure S6. Sequence confirmation of the amplified *Leishmania* spliced-leader target.

Alignment of representative Sanger sequencing reads with the consensus sequence confirmed the identity of the amplified SL-RNA product.

Supplementary File S1: Sanger sequencing reads and available chromatograms.

The archive contains eight untrimmed base-called nucleotide sequences in FASTA format, the original individual sequence files, four available AB1 chromatogram files, and a sequence inventory table. Reads are identified by sample, sequencing primer direction.
